# SGLT2 inhibitor therapy is associated with IL-10/TGF-α/sVEGFR3 signalling and adipose tissue remodelling in advanced heart failure

**DOI:** 10.1016/j.jmccpl.2026.100860

**Published:** 2026-08-16

**Authors:** Anna Cinkajzlova, Barbora Judita Kasperova, Peter Ivak, Ivan Netuka, Vojtech Melenovsky, Lenka Steiner Mrazova, Viktor Stranecky, Milos Mraz, Sona Stemberkova Hubackova, Martin Haluzik

**Affiliations:** a Diabetes Centre, Institute for Clinical and Experimental Medicine, Prague, Czech Republic; b Institute of Medical Biochemistry and Laboratory Diagnostics, First Faculty of Medicine, Charles University and General University Hospital, Prague, Czech Republic; c First Faculty of Medicine, Charles University, Prague, Czech Republic; d Cardiovascular Surgery Department, Institute for Clinical and Experimental Medicine, Prague, Czech Republic; e Third Faculty of Medicine, Charles University in Prague, Prague, Czech Republic; f Cardiology Department, Institute for Clinical and Experimental Medicine, Prague, Czech Republic; g Department of Adipose Tissue Biology, Institute of Physiology of the Czech Academy of Sciences, Prague, Czech Republic; h Research Unit for Rare Diseases, Department of Pediatrics and Inherited Metabolic Disorders, First Faculty of Medicine, Charles University and General University Hospital, Prague, Czech Republic; i Centre for Experimental Medicine, Institute for Clinical and Experimental Medicine, Prague, Czech Republic

**Keywords:** Heart failure, SGLT2 inhibitors, IL-10, TGF-α, sVEGFR3, Adipose tissue remodelling

## Abstract

**Aims:**

Sodium-glucose cotransporter 2 inhibitors (SGLT2i) have demonstrated cardioprotective effects in heart failure (HF), yet the molecular mechanisms underlying these benefits remain incompletely understood. This study aimed to characterize cardioprotective effects of SGLT2i, changes of circulating growth and inflammatory factors, as well as adipose tissue gene expression, in subjects with advanced HF (stage D).

**Methods:**

27 subjects with HF undergoing cardiovascular surgery were included, comprising 17 subjects treated with SGLT2i and 10 untreated controls. Soluble factors were analysed using Luminex assay, and mRNA expression in subcutaneous (SAT) and epicardial adipose tissue (EAT) was assessed.

**Results:**

Subjects receiving SGLT2i exhibited a non-significant trend toward lower BNP concentrations (329.1 ± 78.7 vs. 514.2 ± 103.8 pmol/L, *p* = 0.079) and increased circulatory levels of anti-inflammatory IL-10 (7.2 ± 0.4 vs. 5.8 ± 0.7 pg/mL, *p* = 0.025) and cardioprotective TGF-α (3.5 ± 0.3 vs. 2.7 ± 0.4 pg/mL, *p* = 0.039). sVEGFR3 levels (82.3 ± 4.7 vs. 63.3 ± 3.9 pg/mL, *p* = 0.010) were higher in SGLT2i, suggesting altered lymphangiogenic signalling. Transcriptomic analysis showed lower expression of genes associated with inflammation (*IL6*, *CCL2*, *CXCL2*), cardiac hypertrophy (*PKFP*, *NAMPT*), fibrosis (*TNC*, *TNFAIP3*), and cellular senescence (*ICAM1*, *CDKN1A*), predominantly in SAT.

**Conclusions:**

SGLT2i therapy in advanced HF was associated with higher circulating IL-10, TGF-α and sVEGFR3 levels and with a more favourable adipose tissue gene expression profile characterized by lower expression of genes related to inflammation, fibrosis, and cellular senescence. These findings identify molecular pathways associated with SGLT2i therapy that warrant further mechanistic investigation.

## Highlights

• SGLT2i therapy in advanced HF was associated with higher IL-10, TGF-α, and sVEGFR3 levels.
• Adipose tissue inflammation-related gene expression was lower in advanced heart HF.
• Transcriptomic changes highlights pathways related to reduced fibrosis and senescence.
• SAT was more responsive than EAT to SGLT2i therapy.

## Graphical abstract

**Figure:**
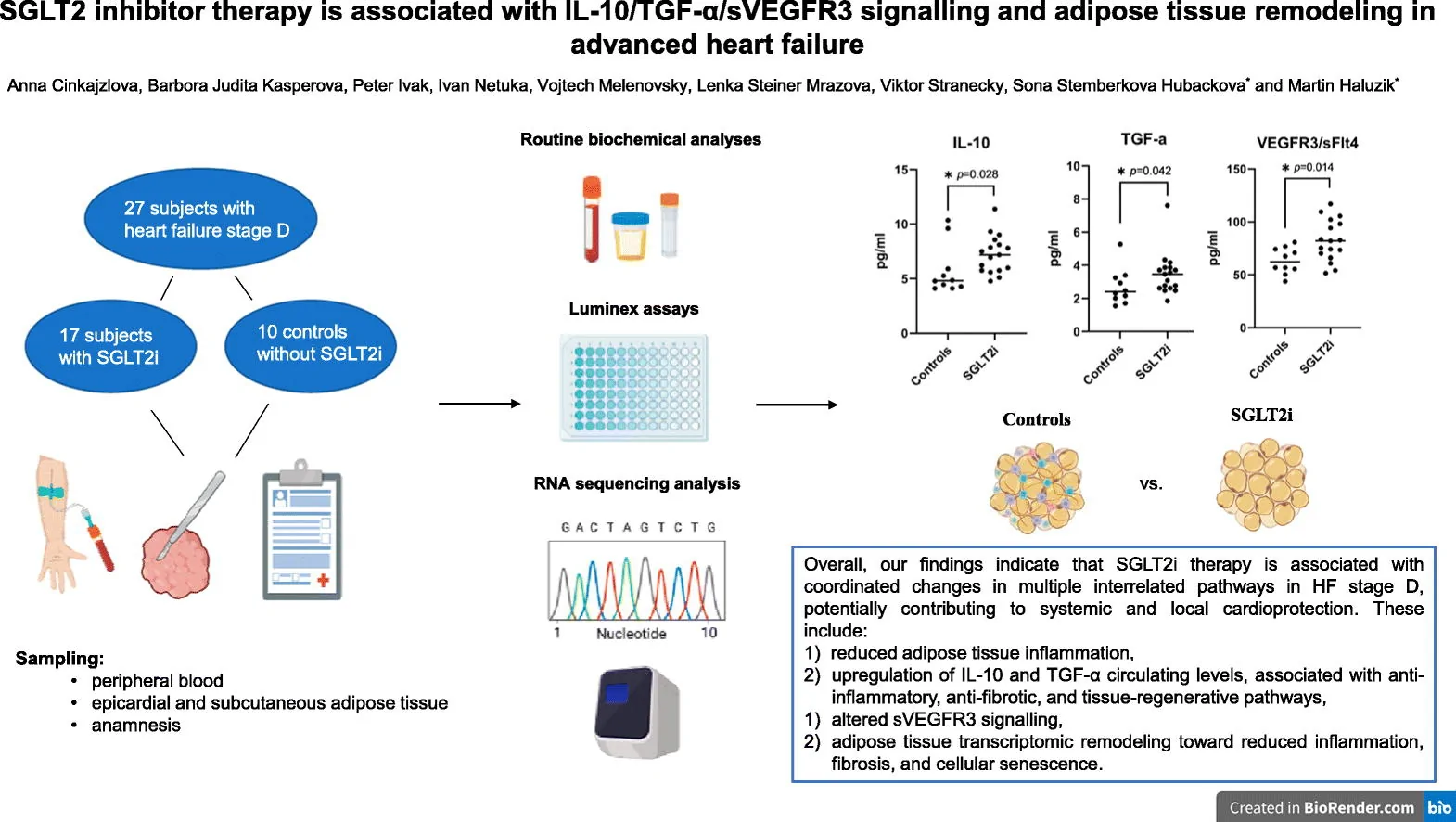

## 1 Introduction

Congestive heart failure (HF) remains a major clinical and public health challenge, representing one of the most severe and complex diseases in modern medicine. According to the definition by the American College of Cardiology and the American Heart Association, it is a complex clinical syndrome that results from any structural or functional impairment of ventricular filling or ejection of blood [1]. In most developed countries, HF is among the leading causes of death, and its prevalence continues to rise, driven primarily by an aging population and advances in medical care that have improved survival in patients with other cardiovascular diseases, allowing more individuals to develop HF over time [2].

Consequently, HF management has become one of the most dynamically developing areas of contemporary medical research and clinical practice. The first option treatment of subjects with reduced ejection fraction includes therapies based on negative influence of renin-angiotensin-aldosterone system (angiotensin receptor-neprysil inhibitors, inhibitors of angiotensin-converting enzyme or angiotensin receptor blockers), β-blockers, antagonists of receptors for mineralocorticoid, diuretics or sodium-glucose co-transporter 2 inhibitors (SGLT2i) [3]. In addition to pharmacological treatment, advanced surgical interventions for HF stage D have expanded considerably. Although heart transplantation remains the standard surgical intervention for end-stage HF [4], the scarcity of donor's hearts has led to a substantial increase in the use of durable left ventricular assist devices (LVADs) over the past two decades. LVADs are designed to maintain adequate cardiac output and restore end-organ perfusion. Major improvements in device durability and a low complications profile have enabled prolonged use, improving both survival and quality of life in affected patients [5], [6], [7].

The SGLT2i treatment based on the blocking of the sodium-dependent glucose transporter 2 located in the early proximal renal tubule represent promising option with multi-system effects management HF across the ejection fraction spectrum, type 2 diabetes mellitus (T2DM), and chronic kidney disease [8]. These drugs achieve anti-hyperglycemic effect through the inhibition of SGLT2 transporter, which is responsible for reabsorbing 80–90% of the glucose filtered by the glomerulus, thereby increasing urinary glucose excretion [9]. This mechanism induces significant caloric loss, body weight reduction, and decrease in blood pressure [10]. In addition, SGLT2i have shown the capacity to reduce major adverse cardiovascular events and hospitalizations for HF [11]. Cardioprotective effects are achieved through the promotion of natriuresis and osmotic diuresis leading to plasma volume contraction and reduced preload along with decrease of blood pressure, arterial stiffness, and afterload. These changes improve subendocardial blood flow in subjects with HF [12]. Another contributing mechanism is promotion of vasodilation and improved endothelial function, along with reduced oxidative stress [12], [13].

Although SGLT2i exert well-described anti-inflammatory and antifibrotic effects, direct evidence for their regulation of specific circulating cardioregulatory proteins remains limited. Among the proteins commonly implicated in cardiac remodelling, fibrosis, and angiogenesis, only a few have been mechanistically linked to SGLT2i action. Preclinical studies demonstrate that SGLT2i can suppress stress-induced up-regulation of endoglin in the myocardium [14], attenuate PDGF family signalling and neointima formation after arterial injury, particularly PDGF-B/BB [15], and dampen high-glucose-induced activation of the VEGF-C/VEGFR3 pathway [16]. More isolated findings suggest possible modulation of galectin-1 in hepatic fibrosis [17]. In contrast, for other factors relevant to cardiovascular injury and repair, including TGF-α, Fas ligand, HB-EGF, IFNγR1, VEGFR1, and SCF (see Table 1 for description of the parameters), current evidences lack the information about regulation by SGLT2i in either experimental or clinical settings. Overall, the influence of SGLT2i on many circulating mediators central to advanced HF pathophysiology remains largely unexplored.

**Table 1:** Selected protein and their associations with cardiovascular system.

Protein	General role	Associations with cardiovascular system	Ref.
BDNF	• regulates synaptic transmission and activity-dependent plasticity	• regulates its development, viability and function • its disturbances in synthesis are related to devastating CV diseases such as high blood pressure and myocardial infarction	[60] [61]
CCL-20/MIP3α	• chemotaxis, Ca^2+^ mobilization, adhesion	• high CCL-20 levels associated with heart ischemic disease, HF and abdominal aortic aneurysm	[62]
Endoglin/CD105	• receptor for TGF-β superfamily • regulation of cell differentiation, migration and adhesion	• promotes the normal balance of VEGF signalling and protect against arteriovenous malformations • soluble form limits TGF-β1 signalling, type I collagen synthesis, and cardiac fibrosis	[63] [64]
Fas ligand/TNFSF6	• member of the TNF family • apoptotic signal	• contributes to final scarring via apoptosis of granulation tissue cells • induces cardiac hypertrophy following pressure overload	[65] [66]
Galectin-1	• cell growth and migration, inflammation, angiogenesis	• promotes angiogenesis and neovascularization through VEGFR2 signalling	[67]
HB-EGF	• cellular proliferation, migration, adhesion, and differentiation	• induces heart fibrosis and proliferation of cardiac fibroblasts	[68] [69]
IFNγR1/CD119	• component of IFN-γ receptor • coordinates immune reaction, cell proliferation, apoptosis	•IFN-γ is a regulator of cardiac hypertrophy in diastolic heart failure •IFN-γ/Stat5 axis may be protective against persistent pressure overload–induced cardiac hypertrophy	[70] [71]
P selectin/CD62P	• cell adhesion through counterligand	• have an action in the initial step of atherosclerosis, marker of plaque stability • increased during coronary artery disease, hypertension and atrial fibrillation	[72] [73]
PDGF-DD	• mitogen for fibroblasts and smooth muscle cells • wound healing, angiogenesis	• its expression increases in the atherosclerotic vessel and infarcted myocardium - promotes scar formation and angiogenesis, stimulates cardiac fibrogenesis (through the TGF-β1) and interacts with the VEGF-A	[74] [75]
SCF/c-kit ligand	• mediate cell survival, migration, and proliferation	• required for regeneration and repair during myocardial damage and vascular repair, improves cardiac function, plays a role in cardiac fibrosis	[76] [77]
TGF-α	• related to EGF and TGF-β • mitogen and neurotrophic factor	• augments mesenchymal stem cell-mediated cardioprotection, stimulates VEGF production and protects cardiac function and reduces infarct size	[78] [43]
VEGFR1/Flt-1	• immune reaction, regulator of macrophage functions	• regulates cardiac remodelling, activates cardiomyocytes, induces angiogenesis and enhances vascular permeability and extracellular proliferation	[79] [80]
VEGFR3/Flt-4	• angiogenesis, vasculogenesis, lymphangiogenesis	• modulates vascular permeability in blood vessel endothelial cells • promote compensatory cardiomyocyte hypertrophy and survival	[81] [82]

Abbreviations: **BDNF**, brain-derived neurotrophic factor; **CCL-20**, C-C motif chemokine ligand 20; **CD**, cluster of differentiation; **CV**, cardiovascular; **EGF**, epidermal growth factor; **HB-EGF**, heparin-binding epidermal growth factor-like growth factor; **HF**, heart failure; **IFN-γ**, interferon gamma; **IFNγR1**, interferon gamma receptor 1; **IL**, interleukin; **MIP3α**, macrophage inflammatory protein-3 alpha; **PDGF-DD**, platelet-derived growth factor-DD; **SCF**, stem cell factor; **TGF-α**, transforming growth factor alpha; **TGF-β**, transforming growth factor beta; **TNF**, tumor necrosis factor; **TNFSF6**, tumor necrosis factor ligand superfamily member 6; **VEGF**, vascular endothelial growth factor; **VEGFR1/Flt-1**, vascular endothelial growth factor receptor 1 (Fms-like tyrosine kinase-1); **VEGFR2**, vascular endothelial growth factor receptor 2; **VEGFR3/Flt-4**, vascular endothelial growth factor receptor 3 (Fms-like tyrosine kinase-4).

In this study, we sought to move beyond the current clinical observations by mapping how SGLT2 inhibition may shape the molecular landscape of advanced cardiovascular disease. By examining a diverse panel of circulating proteins involved in remodelling, fibrosis, angiogenesis, and cardioprotection, our goal was to uncover potential mediators that could explain, and eventually refine the cardioprotective actions of SGLT2i. To complement these systemic markers, we also analysed inflammatory cytokines (IL-6, IL-8, IL-10, and TNF-α) as indicators of subclinical immune activation, together with mRNA expression profiles in EAT and SAT. This integrated approach aims not only to identify promising molecular candidates for future mechanistic studies, but also to open new pathways for understanding how SGLT2i may influence both circulating and tissue-specific signalling in subjects with end-stage heart failure.

## 2 Methods

### 2.1 Study subjects

The study included 27 subjects with advanced HF – 17 subjects treated by SGLT2i and 10 untreated subjects served as a control group. Inclusion criteria comprised a diagnosis of HF stage D [18], a history of HF ≥ 6 months, a left ventricular ejection fraction (LVEF) < 30%, and planned implantation of mechanical circulatory support or heart transplantation due to end-stage HF requiring specialized intervention. All subjects in the SGLT2i group had received treatment for more than 26 days, based on published evidence suggesting the onset of cardioprotective effects after 26 days of therapy [19]. Exclusion criteria for all subjects included acute coronary syndrome, acute inflammatory state and active malignancy.

We conducted a study involving subjects with HF who were scheduled for cardiosurgical interventions. Each subject underwent a rigorous preoperative assessment to ensure optimal surgical outcomes. The etiologies of HF were predominantly categorized as ischemic heart disease or dilated cardiomyopathy, with further patient stratification performed based on detailed anamnestic data (refer to Table 2).

**Table 2:** Baseline characteristics of study subjects.

	Study subjects		Statistics
	Controls	Subjects with SGLT2i	*p* - value
Number (n)	10	17	
SGLT2i therapy (days)	0	217.29 ± 93.61	
Age (year)	59.6 ± 1.8	57.6 ± 2.3	0.545
Gender (m/f)	10/0	15/2	0.516
Diabetics (n; %)	5 (50)	8 (47.1)	1.000
Hypertension (n; %)	5 (50)	14 (82.4)	0.102
Cachexia (n; %)*	3 (33.3)	5 (29.4)	1.000
Atrial fibrillation (n; %)	4 (40)	8 (47.1)	1.000
NYHA class, (n; %)*			
• III	2 (25)	7 (41.2)	
• III-IV	4 (50)	1 (5.9)	
• IV	2 (25)	4 (23.5)	
Left ventricular ejection fraction (%)	20.89 ± 1.85	20.00 ± 0.845	0.922
Left ventricular end-diastolic diameter (mm)	71.778 ± 2.929	67.59 ± 3.24	0.562
Mean pulmonary artery pressure (mmHg)	35.2 ± 4.06	37.53 ± 1.91	0.562
Pulmonary vascular resistance (Woods Units)	4.06 ± 1.294	2.74 ± 0.23	0.732
Mean capillary wedge pressure (mmHg)	22.80 ± 2.118	27.18 ± 2.04	0.030
Cardiac index (l/m^2^/min)	1.908 ± 0.222	1.757 ± 0.178	0.605

Medication			
ACEi/ARb	1	5	0.363
ARNI	6	7	0.440
Β-blockers	9	14	1.000
MRA	8	16	0.535
Digoxin	2	6	0.666
Statins	7	11	1.000

*Not all subjects have NYHA classification assessed. Data are expressed as mean ± SEM. *p < 0.05 controls vs. subjects with SGLT2i treatment; Chi-square test or unpaired *t*-test/Mann-Whitney Rank Sum Test.

Abbreviations: **ACEi**, angiotensin-converting enzyme inhibitor; **ARB**, angiotensin II receptor blocker; **ARNI**, angiotensin receptor–neprilysin inhibitor; **BMI**, body mass index; **HF**, heart failure; **LVEF**, left ventricular ejection fraction; **MRA**, mineralocorticoid receptor antagonist; **NYHA**, New York Heart Association; **SGLT2i**, sodium-glucose cotransporter 2 inhibitor.

All participants signed written informed consent prior to the enrolment into the study. Since the samples were taken as a part of planned surgery, the participants were not compensated. The study was approved by the Human Ethics Review Board, Institute for Clinical and Experimental Medicine (IKEM), Prague, Czech Republic (ethical approval code G-18-36) and was performed in agreement with the principles of WMA Declaration of Helsinki - Ethical Principles for Medical Research Involving Human Subjects [20].

### 2.2 Blood and adipose tissue sampling

Blood samples were taken after 6–12 h of fasting prior to the surgery. Samples were centrifuged for 10 min at 1000 ×*g* within 30 min after withdrawal at room temperature. Plasma or serum aliquots were subsequently stored at −80 °C.

The samples of EAT and SAT were taken perioperatively after 6–12 h of fasting. All procedures were performed from median sternotomy, providing optimal access to the heart. The EAT samples were retrieved from the free wall of right ventricle, in order to avoid potential damage to underlying structures (especially right coronary artery). SAT samples were obtained from predetermined location in the lower pole of the sternotomy site. Aliquots were stored at −80 °C.

### 2.3 Luminex assay

Two independently measured Magnetic bead-based multiplex assays for the Luminex® platform (R&D Systems, Inc., McKinley Place NE, Minneapolis, USA) were used for detection of circulatory levels of selected parameters. A magnetic bead-based 12-plex (LXSAHM-12) was used for determination of TGF-α (sensitivity 0.820 pg/mL), HB-EGF (sensitivity 0.4 pg/mL), IL-8/CXCL8 (sensitivity 1.8 pg/mL), IL-10 (sensitivity 1.6 pg/mL), SCF/c-kit ligand (sensitivity 1.4 pg/mL), CCL-20 (sensitivity 3.4 pg/mL), Fas Ligand/TNSF6 (sensitivity 1.2 pg/mL), P selectin/CD62P (sensitivity 0.9 pg/mL), galectin-1 (sensitivity 632 pg/mL), PDGF-DD (sensitivity 0.200 pg/mL), IFNγR1/CD119 (sensitivity 0.1 pg/mL), and VEGFR3/sFlt-4 (sensitivity 2.7 pg/mL). A magnetic bead-based 6-plex (LXSAHM-06) was used for determination of TNF-α (1.2 pg/mL), IL-6 (1.7 pg/mL), insulin (sensitivity 9.91 pg/mL), BDNF (sensitivity 0.320 pg/mL), Endoglin/CD105 (68.0 pg/mL), and VEGFR1/sFlt-1 (5.68 pg/mL). The intra- and inter-assay variabilities for all assays were between 5.0 and 10.0%.

Routine biochemical parameter and blood count analyses were done at the Laboratory Methods Division, Institute for Clinical and Experimental Medicine (IKEM), Prague, Czech Republic.

### 2.4 RNA Library preparation, sequencing and data analysis

Samples were prepared and analysed as published previously [21] in subgroup of 12 subjects (6 from each study group). Briefly, total RNA was isolated from 80 to 100 mg of adipose tissue samples, using the RNAeasy Lipid Tissue Mini Kit (Qiagen, Hilde, Germany). Its quality was checked using Hs RNA Kit and measured on a 5200 Fragment Analyzer System (Agilent Technologies, Santa Clara, CA, USA). RNA integrity varied among samples (RIN 2.5–7.8), reflecting the clinical nature of surgically obtained adipose tissue. Lower RNA quality was primarily attributable to unavoidable intraoperative factors, including occasional thermal damage from electrocautery and technical challenges during tissue collection, rather than laboratory processing. Because of the RIN variability, the RNA-seq library was prepared by RNA-depletion, using the KAPA RNA HyperPrep Kit with RiboErase (Roche, Rotkreuz, Switzerland), according to the manufacturer's instructions. Libraries were pooled and sequenced on NovaSeq 6000 system (Illumina, San Diego, CA, USA), to produce 100-basepair paired-end reads with average sequencing depth 49 M reads per sample and alignment rate 96% at the National Centre for Medical M - Genomics in Prague.

The resulting FASTQ files were subjected to QC control and trimmed, using Atropos (version1.128). Gene-level abundances were estimated, using Salmon (version 1.3) with the Ensembl gene definition version 94 [22]. Potential technical batch effects were assessed by exploratory analysis of the gene-expression data, including principal component analysis and sample clustering. No substantial batch-related structure was observed, and batch correction was therefore not applied.

Differential expression analysis was performed in R environment (version 4.3.0). The analysis was performed in controls versus subjects with SGLT-2i treatment pairs separately for both EAT and SAT. Groups were compared with the Wald test, and *p*-values were adjusted with the Benjamini–Hochberg method. Resulting differential expression analysis of genes were filtered for significance with soft cut-off criteria, including non-adjusted *p*-value of 0.05, the absolute rounded log2 fold change of 0.5, and the minimal baseMean of 50.

### 2.5 Statistical analysis

**Fig. 1:**
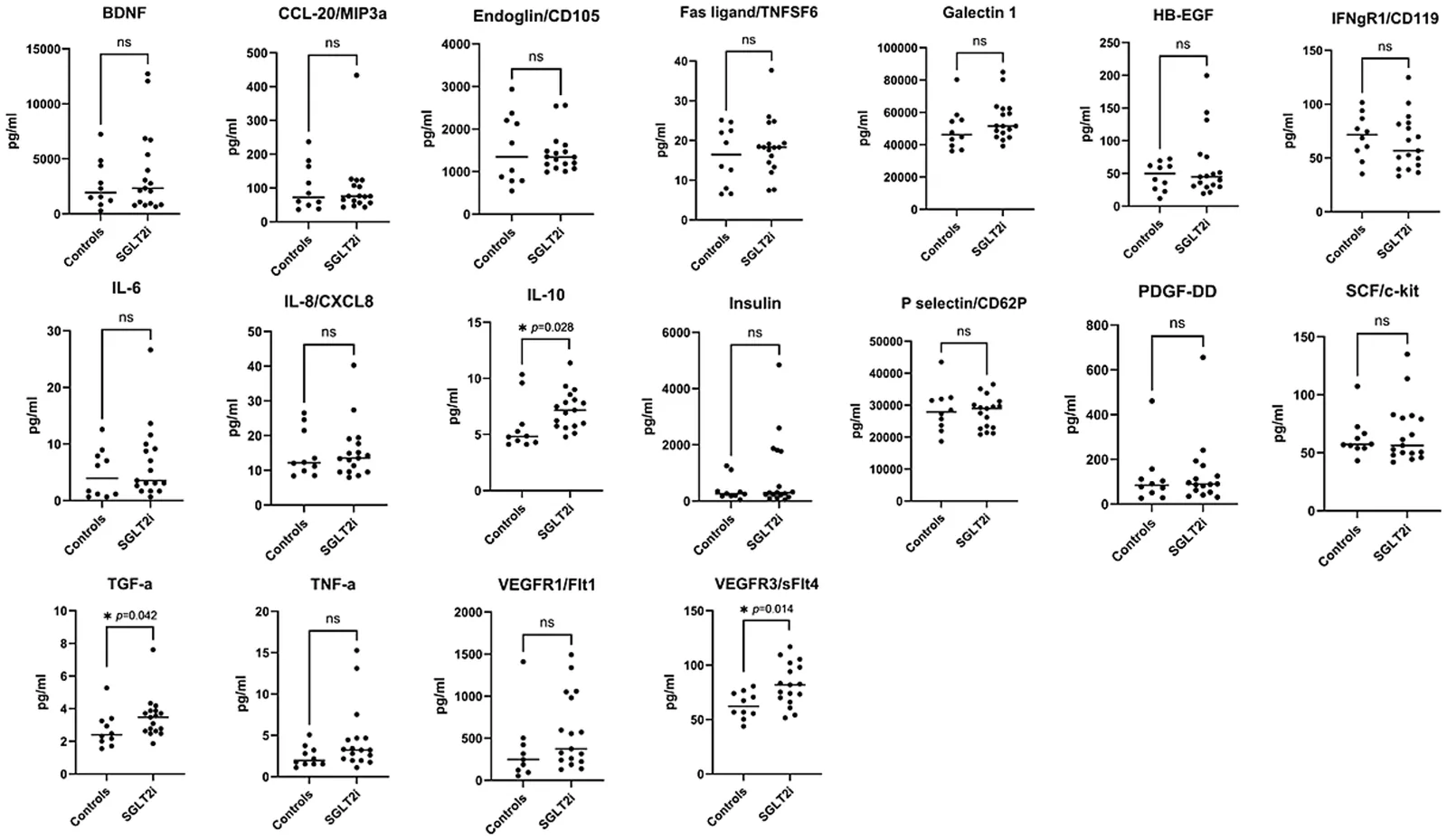
Circulating cytokines, growth factors, and cardioregulatory proteins in subjects with and without SGLT2i treatment. Circulating concentrations of inflammatory mediators, angiogenic, and cardioprotective proteins, and growth factors were measured in control subjects (*n* = 10) and subjects receiving SGLT2i therapy (*n* = 17) by Luminex. Data are presented as individual values with mean ± SEM. *p ≤ 0.05 controls vs. subjects with SGLT-2i treatment; unpaired *t*-test or Mann-Whitney Rank Sum Test, corrected *p* values ≤0.05 are shown. Panels showing statistically significant differences between groups are indicated by an asterisk (*), whereas “ns” denotes non-significant differences. Only IL-10, TGF-α, and VEGFR3/sFlt4 reached statistical significance. All remaining analytes were not significantly different between groups.

Statistical analysis was performed using SigmaPlot 13.0 (SPSS Inc., Chicago, IL, USA) and graphs were created GraphPad Prism 10.2.3 (GraphPad software, Boston, MA, USA). The results are expressed as mean ± standard error of the mean (SEM). Normality of all data was assessed by the Shapiro–Wilk test. Chi-square and Unpaired *t*-test or Mann-Whitney Rank Sum Test were used for the assessment of intergroup differences, as appropriate. Pearson or Spearman correlation test was used to assess the association between biochemical and measured parameters. Statistical significance of all test was assigned to *p* ≤ 0.05. The statistical significance of Luminex and correlation analysis results was adjusted using the Benjamini-Hochberg procedure, with statistical significance defined as a corrected *p* ≤ 0.05. Corrected *p*-values are present in Fig. 1 and data with corrected *p* ≤ 0.05 are shown in Table 3.

**Table 3:** The effect of SGLT2i on biochemical parameters.

	Study subjects		Statistics	Correlation analysis	
	Controls	Subjects with SGLT2i	*p* - value	Positive correlation	Negative correlation
BNP (pmol/L)	514.23 ± 103.81	329.09 ± 78.69	0.079	IL-8 (0.449) IFNγR1 (0.595)	
Hs Troponin T (ng/ml)	110.7 ± 70.5	189.5 ± 110.7	0.744	SCF (0.419) P selectin (0.444) Galectin-1 (0.543)	
BMI (kg/m^2^)	28.59 ± 1.39	28.09 ± 1.12	0.784	Insulin (0.480) BDNF (0.481)	
Glycemia (mmol/L)	6.594 ± 0.826	7.194 ± 0.685	0.451	P selectin (0.470) Galectin-1 (0.397) Insulin (0.429)	
HbA1c (mmol/moll)	44.1 ± 1.8	52.9 ± 3.89	0.138	PDGF-DD (0.451) Insulin (0.504)	
Sodium (mmol/L)	135.63 ± 1.321	135.56 ± 0.709	0.959	HB-EGF (0.430) P selectin (0.409)	Endoglin (−0.461) BDNF (−0.401)
Potassium (mmol/L)	4.174 ± 0.170	4.332 ± 0.101	0.402	TGF-α (0.438)	
Chloride (mmol/L)	102.720 ± 1.307	99.653 ± 1.165	0.105	BDNF (0.387)	Endoglin (−0.461) BDNF (−0.385)
Calcium (mmol/L)	2.203 ± 0.027	2.190 ± 0.042	0.828		
Iron (μmol/L)	13.710 ± 2.112	12.300 ± 1.968	0.752		IL-10 (−0.566) SCF kit (−0.562) IL-6 (−0.426)
Phosphorus (mmol/L)	1.182 ± 0.107	1.219 ± 0.051	0.730	TNF-α (0.424) VEGFR1 (0.532)	
Copper (μmol/L)	18.170 ± 1.702	18.784 ± 1.593	0.958	IL-8 (0.467) CCL-20 (0.503) IFNγR1 (0.564) IL-6 (0.404)	BDNF (−0.430)
Creatinine (μmol/L)	136.8 ± 23.5	117.9 ± 9.1	0.633	SCF kit (0.583) Galectin-1 (0.591) IFNγR1 (0.636)	
Urea (mmol/L)	12.510 ± 2.308	11.341 ± 1.502	0.514	SCF kit (0.459) Galectin-1 (0.535)	BDNF (−0.392)
Uric acid (μmol/L)	369.6 ± 25.0	416.4 ± 41.3	0.424		
Ceruloplasmin (g/L)	0.287 ± 0.021	0.331 ± 0.023	0.210	IL-8 (0.508) CCL-20 (0.523) VEGFR3 (0.385)	
Prealbumin (g/L)	0.203 ± 0.020	0.195 ± 0.016	0.632		IL-8 (−0.566) IL-10 (−0.403) CCL-20 (−0.520) VEGFR3 (−0.415)
Albumin (g/L)	36.580 ± 0.769	35.412 ± 1.263	0.513		IL-10 (−0.447)
Total protein (g/L)	61.450 ± 1.563	64.194 ± 1.893	0.329		
Total bilirubin (μmol/L)	22.930 ± 3.545	21.765 ± 2.707	0.796	Endoglin (0.526)	BDNF (−0.504)
Transferrin (g/L)	2.589 ± 0.092	2.376 ± 0.113	0.207	Endoglin (0.471)	TNF-α (−0.388)
Transferrin saturated (g/L)	21.290 ± 3.447	22.969 ± 3.738	0.874		HB-EGF (−0.484) IL-10 (−0.490) SCF kit (−0.608) CCL-20 (−0.454) IL-6 (−0.414)
Ferritin (μg/L)	209.88 ± 49.464	291.935 ± 77.590	0.880		
Total iron-binding capacity (μmol/L)	65.200 ± 2.323	59.824 ± 2.847	0.205	Endoglin (0.461)	TNF-α (−0.390)
ALT (μkat/L)	0.740 ± 0.161	1.367 ± 0.706	0.393		IFNγR1 (−0.557) IL-6 (−0.414)
AST (μkat/L)	0.640 ± 0.155	0.664 ± 0.165	0.725		IFNγR1 (−0.519)
ALP (μkat/L)	1.823 ± 0.276	1.773 ± 0.242	0.867		Insulin (−0.457)
GGT (μkat/L)	2.543 ± 0.812	1.960 ± 0.297	0.920		
C reactive protein (mg/L)	7.856 ± 3.018	14.941 ± 4.278	0.359	IL-8 (0.463) IL-10 (0.415) VEGFR3 (0.405) TGFα (0.394)	
Total cholesterol (mmol/L)	3.100 ± 0.396	3.547 ± 0.249	0.064	BDNF (0.399)	SCF kit (−0.470) CCL-20 (−0.628) IFNγR1 (−0.566)
HDL cholesterol (mmol/L)	0.819 ± 0.100	0.838 ± 0.051	0.855		
LDL cholesterol (mmol/L)	1.790 ± 0.291	2.029 ± 0.194	0.130	BDNF (0.441)	SCF kit (−0.509) CCL-20 (−0.541)
Triglycerides (mmol/L)	1.105 ± 0.148	1.511 ± 0.180	0.145		
Apolipoprotein B (g/L)	0.655 ± 0.084	0.802 ± 0.063	0.033		SCF kit (−0.510) CCL-20 (−0.473) IFNγR1 (−0.583)
Apolipoprotein A1 (g/L)	0.950 ± 0.094	0.962 ± 0.051	0.905		
TSH (mIU/L)	4.038 ± 0.774	4.268 ± 1.058	0.651		
T3 free (pmol/L)	3.288 ± 0.286	3.244 ± 0.193	0.895		IFNγR1 (−0.557)
T4 free (pmol/L)	14.239 ± 0.494	15.267 ± 0.671	0.294		
Cortisol (nmol/L)	579.180 ± 151.607	349.006 ± 38.566	0.292	IL-8 (0.556) P selectin (0.507) PDGF (0.407)	
eGRF (ml/s/1.73 m^2^)	1.005 ± 0.154	1.048 ± 0.089	0.796		SCF (−0.598) Galectin-1 (−0.682) IFNγR1 (−0.628)

Data are expressed as mean ± SEM. *p < 0.05 controls vs. subjects with SGLT2i treatment; unpaired t-test or Mann-Whitney Rank Sum Test. Pearson or Spearman correlation test was used to assess the association between biochemical and measured parameters, the results were corrected by Benjamini–Hochberg procedure, only results with corrected *p* < 0.05 are mentioned, R value is shown in parentheses.

Abbreviations: **ALT**, alanine aminotransferase; **AST**, aspartate aminotransferase; **BNP**, B-type natriuretic peptide; **eGFR**, estimated glomerular filtration rate; **GGT**, gamma-glutamyl transferase; **HbA1c**, glycated hemoglobin; **HDL**, high-density lipoprotein; **hs Troponin T**, high-sensitivity troponin T; **LDL**, low-density lipoprotein; **SGLT2i**, sodium-glucose cotransporter 2 inhibitor.

## 3 Results

### 3.1 Baseline characteristics of study subjects

Subjects (*n* = 27) undergoing heart transplantation or the implantation of a LVAD for HF stage D were divided according to SGLT2i medication on group without SGLT2i (10 subjects) and group with SGLT2i long-term treatment (17 subjects, 10 subjects with dapagliflozin, 7 subjects with empagliflozin, all in minimal 10 mg daily-dose). The mean age of subjects in both groups was approximately 60 years. Baseline demographic, clinical, hemodynamic, and echocardiographic characteristics, as well as concomitant pharmacotherapy, were generally comparable between the groups. The prevalence of diabetes, hypertension, cachexia and atrial fibrillation was comparable between groups, with no significant differences in left ventricular ejection fraction (LVEF), left ventricular end-diastolic diameter, mean pulmonary artery pressure, pulmonary vascular resistance, or cardiac index. The higher pulmonary capillary wedge pressure observed in the SGLT2i group represents a baseline imbalance and is acknowledged as a limitation of the study (Table 2).

### 3.2 Effects of SGLT2i treatment on cardiovascular and biochemical parameters

In our study cohorts, subjects with SGLT2i treatment have higher levels of apolipoprotein B concentrations together with non-significant trend toward higher total cholesterol levels compared to subjects without SGLT2i (Table 3). A non-significant trend toward lower B-type natriuretic peptide (BNP) concentrations was also observed in the SGLT2i group (514.2 ± 103.8 vs. 329.1 ± 78.7 pmol/L, *p* = 0.079), which is consistent with the literature [23]. No significant differences or non-significant trends were detected in the remaining biochemical, lipid, or metabolic parameters between the study groups (Table 3).

### 3.3 The SGLT2i treatment was associated with changes in circulating anti-inflammatory and cardioprotective cytokines

The SGLT2i treatment resulted in increased IL-10 levels (Fig. 1), which is the main anti-inflammatory cytokine and could be associated with SGLT2i induced macrophage polarization toward M2 observed in preclinical studies [24]. A concurrent increase in transforming growth factor-α (TGF-α) was also observed (Fig. 1), and a significant positive correlation between IL-10 and TGF-α levels was identified (*R* = 0.555, *p* = 0.003, *p*^*c*^ = 0.012). TGF-α itself is known to exert beneficial effects on the myocardium, including promotion of tissue repair and protection against injury [25]. In addition, subjects with SGLT2i exhibited higher circulating concentrations of soluble VEGFR3, factor regulating angiogenesis, tissue repair, and inflammation, which also positively correlated with both; IL-10 (*R* = 0.605, *p* = 0.001, *p*^*c*^ = 0.004) and TGF-α (*R* = 0.440, *p* = 0.022, *p*^*c*^ = 0.016).

### 3.4 SGLT2i treatment is associated with favourable adipose tissue transcriptomic remodelling

Interestingly, consistent with the notion that SGLT2i exert prominent metabolic effects within EAT and SAT, we observed a significant reduction in the expression of several genes linked to the cardiac hypertrophy or dysfunction, including *PFKP*, *RELB*, *NAMPT*, *DUSP4* and *ANGPT2* in subjects receiving SGLT2i treatment. Lower expression of genes related to cellular stress and senescence (*GADD45B*, *CDKN1A*, *THBS1*, and *ICAM1*) was also observed, although differences for these transcripts represented non-significant trends. This suggests a potential attenuation of adipose tissue inflammation and aging [26], [27]. While expression of pro-inflammatory genes declined in both adipose depots, the reduction in *IL6*, *CCL2*, *CXCL2*, *IL1R1*, and *IL1RAP* reached statistical significance only in SAT. Importantly, *TNC* (Tenascin-C) and *TNFAIP3*, genes linked to regulation of fibrosis, were significantly downregulated in both SAT and EAT. Notably, mRNA expression of leptin receptor (*LEPR*) was significantly increased in EAT from subjects receiving SGLT2i therapy (Table 4).

**Table 4:** The effect of SGLT2i on mRNA expression in epicardial and subcutaneous adipose tissue.

	Symbol	Gene name	EAT		SAT	
			Log2 fold change	*p* - value	Log2 fold change	*p* - value
Inflammation	*IL6*	interleukin 6 (interferon, beta 2)	−0.685	0.446	**−3.419**	**0.001**
	*CCL2*	chemokine (C-C motif) ligand 2	−0.214	0.505	**−2.645**	**0.001**
	*CSF2RA*	colony stimulating factor 2 receptor, alpha, low-affinity (granulocyte-macrophage)	−0.716	0.068	–	–
	*CXCL2*	chemokine (C-X-C motif) ligand 2	−0.700	0.166	**−1.710**	**0.035**
	*IL1R1*	interleukin 1 receptor type 1	–	–	**−1.424**	**0.004**
	*IL1RAP*	interleukin 1 receptor accessory protein	–	–	**−1.333**	**0.017**
	*TRAF1*	TNF receptor associated factor 1	−0.397	0.286	–	–
	*ITGAM*	integrin subunit alpha M	−0.462	0.195	–	–
	*ITK*	IL2 inducible T-cell kinase	−0.666	0.101	–	–
	*SELE*	selectin E	−0.206	0.842	**−2.013**	**0.037**
	*RELT*	RELT tumor necrosis factor receptor	−0.242	0.433	**−1.227**	**0.044**
Metabolism	*RYR2*	ryanodine receptor 2	−1.022	0.271	–	–
	*ATP1B3*	ATPase Na+/K+ transporting subunit beta 3	−0.349	0.472	−1.442	–
	*FADS1*	fatty acid desaturase 1	−0.342	0.367	**−1.813**	**0.005**
	*GLUL*	glutamate-ammonia ligase	−0.186	0.556	**−1.465**	**0.001**
	*IRS1*	insulin receptor substrate 1	−0.232	0.446	–	–
	*LDLR*	low density lipoprotein receptor	−0.410	0.322	**−1.277**	**0.041**
	*LPIN1*	lipin 1	−0.502	0.080	**−2.045**	**0.000**
	*MAT2A*	methionine adenosyltransferase 2A	−0.252	0.459	**−1.357**	**0.006**
	*PGM3*	phosphoglucomutase 3	−0.140	0.682	**−1.584**	**0.000**
	*PNP*	purine nucleoside phosphorylase	−0.411	0.230	**−2.282**	**0.003**
	*UPP1*	uridine phosphorylase 1	−0.205	0.593	**−1.599**	**0.022**
	*LEPR*	leptin receptor	**1.170**	**0.004**	0.295	0.437
Cellular senescence and stress	*ICAM1*	intercellular adhesion molecule 1	−0.647	0.133	−1.337	0.066
	*CDKN1C*	cyclin-dependent kinase inhibitor 1C (p57, Kip2)	−0.166	0.647	–	–
	*CDKN1A*	cyclin-dependent kinase inhibitor 1A	–	–	−1.306	0.060
	*DUSP4*	dual specificity phosphatase 4	−0.114	0.809	**−1.207**	**0.030**
	*GADD45B*	growth arrest and DNA-damage-inducible, beta	−0.210	0.638	−1.251	0.056
Fibrosis/cardiac remodelling	*CXCR4*	chemokine (C-X-C motif) receptor 4	−0.516	0.293	–	–
	*IGF1*	insulin like growth factor 1	−0.353	0.207	**−1.673**	**0.000**
	*TNFAIP3*	TNF alpha induced protein 3	**−1.274**	**0.017**	**−1.728**	**0.025**
	*COL1A1*	collagen type I alpha 1	−0.446	0.174	–	–
	*COL3A1*	collagen type III alpha 1	−0.115	0.719	–	–
	*COL4A1*	collagen type IV alpha 1	−0.192	0.565	–	–
	*FN1*	fibronectin 1	−0.300	0.405	–	–
	*TNC*	tenascin C	**−1.759**	**0.015**	**−2.407**	**>0.001**
	*THBS1*	thrombospondin 1	−0.309	0.494	−1.413	0.053
	*BIRC3*	baculoviral IAP repeat containing 3	−0.225	0.589	**−1.991**	**0.002**
	*CREB3L1*	cAMP responsive element binding protein 3-like 1	−0.219	0.375	**−1.472**	**0.002**
	*SFRP4*	secreted frizzled-related protein 4	−0.125	0.850	−0.110	0.877
Cardiac hypertrophy, dysfunction	*NFATC2*	nuclear factor of activated T-cells, cytoplasmic, calcineurin-dependent 2	−0.221	0.415	–	–
	*ANGPT2*	angiopoietin 2	−0.243	0.458	**−2.014**	**0.001**
	*NAMPT*	nicotinamide phosphoribosyltransferase	−0.121	0.789	**−1.681**	**0.006**
	*PFKP*	phosphofructokinase, platelet	−0.535	0.057	**−1.492**	**0.002**
	*RELB*	v-rel avian reticuloendotheliosis viral oncogene homolog B	−0.124	0.688	**−1.210**	**0.048**

Data are expressed as mean ± SEM. *p < 0.05 subjects with SGLT2i vs controls, treatment; unpaired *t*-test or Mann-Whitney Rank Sum Test, – the expression was not determined. The significance of bold is statistical significance with *p* ≤ 0.05.

## 4 Discussion

HF is a multifactorial clinical syndrome characterized by impaired cardiac function and structure, leading to inadequate cardiac output, compensatory neurohormonal activation and increased left ventricular filling pressure [28]. In this study, we investigated circulating proteins and adipose tissue genes involved in cardiovascular remodelling and evaluated their association with SGLT2i therapy in subjects with advanced HF (stage D).

Consistent with previous studies [28], [29] our findings are compatible with the established cardioprotective effects of SGLT2i. Although both patient groups had comparable BMI and similar prevalence of diabetes, cachexia, and hypertension, subjects receiving SGLT2i exhibited a non-significant trend toward lower BNP concentrations. SGLT2i are also known to exert anti-inflammatory actions, which are associated with cardio- and nephroprotective effects. We observed elevated IL-10 levels in SGLT2i-treated subjects. This finding supports experimental data showing that dapagliflozin increases myocardial IL-10 and reduces collagen synthesis through activation of anti-inflammatory macrophages and inhibition of myofibroblast differentiation after myocardial infarction [30]. Similarly, empagliflozin has been shown to promote M2 macrophage polarization and adipose tissue browning [31]. IL-10 is also known to exert cardioprotective effects, including reducing infarct size, improving cardiac function, enhancing capillary density [32], and limiting apoptosis and inflammation via heme clearance and STAT3 inhibition [33]. The higher circulating IL-10 concentrations observed in our cohort are therefore consistent with these experimental findings.

Interestingly, IL-10 levels positively correlated with TGF-α levels. TGF-α is an essential cardioprotective factor that enhances mesenchymal stem cell-mediated cardioprotection during acute ischemia and reperfusion in isolated heart models [34]. In addition, it has been shown that macrophages present in wounds express TGF-α antigen, while wound fluids themselves contain TGF-α. TGF-α may mediate angiogenesis, epidermal regrowth, and the formation of granulation tissue in vivo [35]. Molecular background of TGF-α mediated protection comprises more mechanisms. TGF-α suppresses local IL-1β and IL-6 production, both mediators of myocardial dysfunction [36], and enhances fibroblast-mediated extracellular matrix turnover through MMP-2 activation [34]. In addition, TGF-α upregulates vascular endothelial growth factor (VEGF), linking anti-inflammatory signalling with angiogenic remodelling [37].

VEGF protein plays a crucial role in ischemia-induced angiogenesis and is considered as a biomarker of HF mortality. The VEGF family consists of VEGF-A, VEGF-B, VEGF-C, VEGF-D, and placenta growth factor. Each isoform has a distinct biological function and exert its effect by binding to specific tyrosine kinase receptors known as vascular endothelial growth factor receptors (VEGFRs). VEGFR1, and VEGFR2 are expressed on the vascular endothelial cells, and VEGFR3 is expressed on the lymphatic endothelial cells [38]. Their soluble forms regulate angiogenesis, vascular permeability, and endothelial function, and dysregulation of these pathways has been closely linked to the onset and progression of cardiovascular diseases. In our cohort, circulating concentrations of sVEGFR3 were significantly higher in subjects receiving SGLT2i therapy. While activation of VEGFR1 supports angiogenesis and has anti-apoptotic and antioxidative effects, VEGFR3 enhances lymphangiogenesis, fibrosis, and inflammation [38] and also impacts cell survival, calcium flux, vessel permeability, and collecting lymphatic vessel contractility [39]. Conversely, elevated VEGFR3 levels may also exert protective effects. VEGF-C/VEGFR3 signalling has been shown to limit the transition from cardiac hypertrophy to heart failure by preserving lymphatic drainage and tissue fluid homeostasis [40]. In addition, sVEGFR3 has been reported to attenuate myocardial ischaemia/reperfusion injury by inhibiting mitochondria-driven apoptosis, reducing the proportions of M1 macrophages and T helper cells in myocardial tissue, and decreasing the production of their key pro-inflammatory cytokines TNF-α and IFN-γ [41]. However, the biological effects of sVEGFR3 may be context-dependent, as excessive circulating levels could sequester VEGF-C and thereby restrict sVEGFR3-mediated lymphangiogenesis and adversely affect vascular permeability [42]. Overall, the increase in sVEGFR3 observed in subjects receiving with SGLT2i may represent a compensatory response supporting endothelial and lymphatic homeostasis during HF progression. This response may also be linked to the tissue-regenerative effects of TGF-α, which can promote VEGF production [43] and to M2-like macrophage polarization associated with IL-10 production [44]. The positive correlations observed among circulating TGF-α, IL-10, and sVEGFR3 levels support a potential interplay between these pathways, although the observational nature of our study precludes conclusions regarding causal or mechanistic relationships.

Adipose tissue actively communicates with the cardiovascular system through endocrine and paracrine signalling [45]. Adipose tissue depots are functionally and morphologically heterogeneous, acting as sources of bioactive molecules that regulate the physiological functioning of the vasculature and myocardium [46]. EAT contributes to the development and progression of HF by modulating adipogenesis, cardiac remodelling, insulin resistance, cardiac function, and the renin–angiotensin–aldosterone system, as well as by secreting a wide range of bioactive molecules involved in the regulation of inflammatory responses [47]. While prior studies suggested stronger SGLT2i effects on EAT or VAT due to more pronounced depot reduction [48], our RNA sequencing revealed a greater transcriptomic impact in SAT, what is in line of our previous results [21]. The reasons for this apparent depot-specific response remain uncertain. Adipose tissue depots are functionally and molecularly heterogeneous, and their responsiveness may differ according to the local microenvironment and stage of disease. In subjects with advanced stage D HF, EAT may already be subject to extensive disease-related structural and inflammatory remodelling, potentially limiting the detection of additional treatment-associated transcriptional differences. In contrast, SAT may retain greater metabolic and transcriptional plasticity, allowing treatment-associated changes to be more readily detected. However, differences in tissue sampling and the limited number of subjects included in the RNA-sequencing analysis may also have contributed to the apparent predominance of changes in SAT. Therefore, our findings should not be interpreted as evidence that SAT is intrinsically more responsive to SGLT2i than EAT. SGLT2i treatment was associated with downregulation of the inflammatory genes including *IL6*, *CCL2*, *CXCL2* or *IL1R1* consistent with an anti-inflammatory shift, while lower *CCL2* and *CXCL2* expression indicates decrease in monocyte and neutrophil recruitment [49], [50]. Similarly, lower expression of genes associated with cardiac hypertrophy (*PFKP*, *NAMPT*) and cardiac dysfunction (*RELB*, *ANGPT2*, *DUSP4*) in SAT may reflect molecular changes associated with pathways involved in cardiac remodelling rather than direct effects of SGLT2i therapy. As hypertrophy precedes HF and contributes to contractile impairment [51], this transcriptional pattern supports cardioprotective potential of SGLT2i.

SGLT2i treatment was further associated with reduced *ICAM1* and *CDKN1A* expression in SAT, suggesting attenuation of cellular senescence, which is related to inflammation as well as metabolic and endocrine changes observed in HF [26], [27]. Senescent cells, though metabolically active, secrete pro-inflammatory and pro-fibrotic molecules collectively named senescence-associated secretory phenotype that drive tissue damage, chronic inflammation, fibrosis, and metabolic dysfunction [52]. Reduced expression of these genes may indicate altered adipose tissue homeostasis associated with attenuation of cachexia or diabetes, although functional confirmation was beyond the scope of the present study. Our transcriptomic data also revealed reduced expression of fibrotic mediators *Tenascin C* (*TNC*) and *TNFAIP3* in both SAT and EAT. TNFAIP3 negatively regulates toll-like receptor (TLR)-mediated fibrotic signalling by inhibiting polyubiquitination of pathway intermediates [53]. Although its downregulation could theoretically weaken protection, basal TNFAIP3 levels are low in healthy tissue, and reduced expression may indicate lower inflammatory activation [54]. TNC, typically low in adult myocardium, is upregulated during cardiac injury and fibrosis [55]. It promotes fibroblast activation and extracellular matrix stabilization via TLR4 signalling [56]. Moreover, TNC interacts with VEGF-A to regulate endothelial proliferation [57] and lymphangiogenesis [58]. Lower TNC expression is therefore compatible with reduced extracellular matrix remodelling, although functional consequences cannot be inferred from transcriptomic data alone. Interestingly, IL-10, elevated in our SGLT2i group, can inhibit TNC expression, suggesting a mechanistic link between anti-inflammatory signalling and reduced matrix remodelling [59].

Overall, our findings point to a potential interplay between systemic cardioregulatory signalling and adipose tissue remodelling in subjects with advanced HF receiving SGLT2i therapy. The observed associations suggest that the effects of SGLT2i may extend beyond their established haemodynamic and metabolic actions and involve coordinated changes in inflammatory, regenerative, vascular, and tissue-remodelling pathways. The parallel alterations in circulating mediators and adipose tissue gene expression further highlight adipose tissue as a potentially relevant component of the systemic response to SGLT2 inhibition in advanced HF. Given the observational and cross-sectional design of the study, these findings should be interpreted as hypothesis-generating and provide a rationale for future studies investigating the functional interactions among these pathways and their contribution to the cardiovascular benefits of SGLT2i.

## 5 Conclusions

In the present study, SGLT2i therapy in subjects with advanced HF was associated with higher circulating IL-10, TGF-α, and sVEGFR3 levels, and adipose tissue transcriptomic changes characterized by lower expression of genes related to inflammation, fibrosis, and cellular senescence. These findings identify candidate molecular pathways associated with SGLT2i therapy in advanced HF but do not establish causal mechanisms. Therefore, the proposed roles of the IL-10/TGF-α/sVEGFR3 axis and adipose tissue remodelling should be regarded as hypothesis-generating and require confirmation in larger prospective and mechanistic studies.

### 5.1 Limitations

This study has several limitations. First, it was performed in a relatively small cohort of subjects with advanced (stage D) heart failure undergoing cardiovascular surgery at a single centre. The unequal group sizes and the limited number of samples available for RNA sequencing reduced statistical power and may have increased the risk of both type I and type II errors. Consequently, borderline statistical differences should be interpreted cautiously and regarded as hypothesis-generating rather than definitive evidence of biological effects. The transcriptomic analysis should be considered exploratory, as candidate transcripts were selected based on unadjusted rather than FDR-corrected p-values, increasing the risk of false-positive findings. In addition, RNA integrity varied among samples because adipose tissue was obtained during complex cardiovascular surgical procedures, and tissue quality was occasionally affected by unavoidable intraoperative factors, including thermal damage related to electrocautery and technical challenges during tissue collection. RNA integrity varied (RIN 2.5–7.8) and was not included as a covariate in the differential expression model. Furthermore, the limited amount of surgically obtained adipose tissue precluded independent validation of the RNA sequencing results by qPCR or immunohistochemistry, and therefore the transcriptomic findings should be considered exploratory until confirmed in independent cohorts.

Because this was an observational cross-sectional study, causality cannot be established. Although baseline pharmacological therapy has been reported in detail, the relatively small cohort size and the observed baseline imbalances, including differences in pulmonary capillary wedge pressure, precluded meaningful multivariable regression or propensity score analyses, as these approaches would be underpowered and susceptible to overfitting. Consequently, residual confounding by concomitant medications, comorbidities, disease severity, baseline clinical differences, or other clinical factors cannot be excluded. Finally, the transcriptomic findings require validation in larger independent cohorts.

## CRediT authorship contribution statement

**Anna Cinkajzlova:** Writing – original draft, Investigation, Formal analysis, Data curation. **Barbora Judita Kasperova:** Writing – original draft, Formal analysis, Data curation. **Peter Ivak:** Writing – review & editing, Project administration. **Ivan Netuka:** Writing – review & editing, Investigation, Data curation. **Vojtech Melenovsky:** Writing – review & editing, Investigation. **Lenka Steiner Mrazova:** Investigation, Formal analysis, Data curation. **Viktor Stranecky:** Investigation, Formal analysis, Data curation. **Milos Mraz:** Writing – review & editing. **Sona Stemberkova Hubackova:** Writing – review & editing, Methodology, Investigation, Conceptualization. **Martin Haluzik:** Writing – review & editing, Supervision.

## Ethics approval and consent to participate

The study was conducted in accordance with the World Medical Association Declaration of Helsinki - Ethical Principles for Medical Research Involving Human Subjects as revised in 2013, and approved by the Human Ethics Review Board, Institute for Clinical and Experimental Medicine, Prague, Czech Republic (ethical approval code G-18-36).

## Funding

This work was supported by grants from the Czech Ministry of Health (NV19-02-00118 and NU20-02-00190), the 10.13039/501100001824Czech Science Foundation (25-15252S), by the project National Institute for Research of Metabolic and Cardiovascular Diseases (Programme EXCELES, ID Project No. LX22NPO5104) - Funded by the 10.13039/501100000780European Union – Next Generation EU, RVO VFN 64165 and by CZ - DRO (“Institute for Clinical and Experimental Medicine – IKEM, IN 00023001”).

## Declaration of competing interest

The authors declare that they have no known competing financial interests or personal relationships that could have appeared to influence the work reported in this paper.
